# Fast calculation of hydrogen-bond strengths and free energy of hydration of small molecules

**DOI:** 10.1038/s41598-023-30089-x

**Published:** 2023-03-13

**Authors:** Gian Marco Ghiandoni, Eike Caldeweyher

**Affiliations:** 1 Augmented DMTA Engineering, R&D IT, AstraZeneca, Eastbrook House, Shaftesbury Road, Cambridge, CB2 8DU UK; 2 Augmented DMTA Engineering, R&D IT, AstraZeneca, Pepparedsleden 1, 43183 Mölndal, Sweden

**Keywords:** Virtual screening, Cheminformatics, Cheminformatics

## Abstract

Hydrogen bonding is an interaction of great importance in drug discovery and development as it may significantly affect chemical and biological processes including the interaction of small molecules with other molecules, proteins, and membranes. In particular, hydrogen bonding can impact drug-like properties such as target affinity and oral availability which are critical to developing effective pharmaceuticals, and therefore, numerous methods for the calculation of properties such as hydrogen-bond strengths, free energy of hydration, or water solubility have been proposed over time. However, the accessibility to efficient methods for the predictions of such properties is still limited. Here, we present the development of Jazzy, an open-source tool for the prediction of hydrogen-bond strengths and free energies of hydration of small molecules. Jazzy also allows the visualisation of hydrogen-bond strengths with atomistic resolution to support the design of compounds with desired properties and the interpretation of existing data. The tool is described in its implementation, parameter fitting, and validation against two data sets of experimental hydration free energies. Jazzy is also applied against two chemical series of bioactive compounds to show that hydrogen-bond strengths can be used to understand their structure–activity relationships. Results from the validations highlight the strengths and limitations of Jazzy, and suggest its suitability for interactive design, screening, and machine-learning featurisation.

## Introduction

Hydrogen bonding plays a key role in the natural world due to its ubiquitous presence. It is responsible for many of the properties of water that are fundamental to life and represents the most significant type of non-covalent interaction in biological systems resulting in phenomena such as base-pair formation in the DNA double helix, protein folding, and molecular recognition^1–3^.

Hydrogen bonding also affects the interactions of small-molecule drugs at different levels of complexity, going from those with other small molecules up to the highest supramolecular assemblies, e.g., proteins and membranes^4^. These interactions may significantly impact the biological activity, pharmacokinetics, and physicochemical properties of drugs, hence making hydrogen bonding an important subject of study in drug discovery and development^5^. In particular, understanding this topic is key to the design of orally available drugs, which remains a major challenge in pharmaceuticals as achieving optimal bioactivity and bioavailability often involves balancing lipophilicity and water solubility^6–8^. Interestingly, the lack of such a balance has been found to be a prime factor for the attrition of highly potent compounds in both pre-clinical and clinical development stages, hence suggesting that the evaluation of drug-like properties should follow more rigorous criteria^9^.

Drug-like properties are often predicted using computational methods to reduce the number of cycles necessary to obtain candidates with suitable profiles. Several methods for the estimation of hydrogen-bond strengths and water solubility have been proposed in the last decades, ranging from cut-off guidelines (e.g., the “rule of five”^10^) to highly accurate quantum mechanics models for the quantitative prediction of free energies of hydration^11–17^. However, despite the large variety of techniques described in the literature, the accessibility to efficient methods for the prediction of such properties is still restricted.

Herein, we present an open-source reimplementation of the method proposed by Gerber for calculating hydrogen-bond strengths and free energy of hydration of molecules^18^. Our tool, referred to as Jazzy, relies on the calculation of atomic partial charges and van der Waals radii from a molecule conformation using the method proposed by Caldeweyher, which are then used to produce three contribution terms to the free energy of hydration^19,20^. These terms, namely, polar, apolar, and interaction, are finally summed up to yield the total free energy of hydration. Hydrogen-bond strengths are calculated as part of the process at both the atomic and molecular levels. Strengths and free energies can be either used for screening purposes or as features for modelling more complex molecular properties including pharmacokinetics. In addition, Jazzy enables the visualisation of atomic hydrogen-bond strengths as molecule renderings, where donors and acceptors are labelled and highlighted with different colour gradients, to support the design of compounds with desired properties or the interpretation of existing data.

We have reported the details of the implementation of Jazzy, parameter fitting, and validation against two data sets of hydration free energies from Gerber’s and Guthrie’s and colleagues’ works^21^. We have also described the retrospective application of our method in medicinal chemistry: First, we have applied Jazzy against a chemical series of inhibitors from Chen et al.^22^, which were suggested to share the same donor interactions with the receptor, and have shown that our method can support understanding the structure–activity relationship of compounds. Second, we have applied Jazzy against a series of inhibitors from Robb et al.^23^, which were suggested to share the same acceptor interactions with the target, and shown that our method produced results correlated with both experimental activities and acceptor strengths calculated with a quantum mechanics-based method. The simplicity of Jazzy does not allow modelling solvent, intramolecular, and supramolecular effects; however, its implementation enables the calculation of hundreds of structures per minute on a standard laptop, and the results presented in this publication suggest that it can be used as an alternative to heavy computational tools in drug discovery and development. The use of Jazzy for molecular modelling using machine learning will be described in a future publication.

## Implementation

Jazzy is a simplified reimplementation of the method described by Gerber, where the Free Energy of Hydration of a small molecule is given as the sum of three quantities, namely, polar, apolar, and interaction terms. Hydrogen-bond strengths are generated as adimensional values as a part of the calculation process as described as follows. Jazzy depends on kallisto^19^, an open-source method proposed by Caldeweyher for the calculation of partial charges and other quantum mechanical features. The electronegativity equilibration equations used to calculate partial charges in kallisto incorporate atomic parameters which were fitted to reproduce PBE0/def2-TZVP Hirshfeld partial charges^24^. The selection of kallisto was motivated by its accuracy, speed, and licensing model. In addition, the calculation of charges from kallisto has also been shown to be applied effectively to physical modelling including the correction of London dispersion in density functional theory^24^. The source code, fitting, validation, and usage of Jazzy can be found in the repository https://github.com/AstraZeneca/jazzy. The version of Jazzy described in this work was implemented in Python 3.8, uses RDKit 2021.09.04^25^ and kallisto 1.0.7.

### Polar term

Our method consists of calculating the partial charges of a molecule using kallisto to produce hydrogen-bond donor of hydrogens and acceptor strengths of atoms with lone pairs according to Eqs. (1) and (2), then using those strengths to derive the polar contribution to the hydration free energy. As shown in the equations, the donor (sd) strength is obtained by summing the partial charges of a hydrogen (qH) to a corrective term (δqH), and the acceptor (sa) strength comes from summing the partial charges of an atom with lone pairs (qa) to another corrective term (δqa). Both corrective terms (δqH and δqa) account for the influence of charges from neighbour atoms as shown in Eq. (3). The donor and acceptor sums are then adjusted by multiplying them against coefficients (D and A) obtained from the calibration of our method to yield donor and acceptor strengths equal to 1.0 for the hydrogens and oxygen in a water molecule, respectively. The calibration against water was deliberate as it facilitates the understanding of those strengths when analysing compounds in biological systems, i.e., atoms with strengths greater than 1.0 can form hydrogen bonds that are stronger than those formed by a water molecule and vice versa. D and A are set to 63.7 and − 4.4362 for a water molecule minimised using the MMFF94 method implemented in RDKit. While Eq. (1) is identical to that described by Gerber, Eq. (2) was intentionally simplified by removing the hybridization dipole (p_hi_), quadrupole moment (w_i_), and the corrective term (A_0_) defined in the original paper. These modifications were introduced to increase the performance and generalisability of the model, hence resulting in a simplified reimplementation.

1 $$sd=D\left(qH + \delta qH\right)$$

2 $$sa=A\left(qa + \delta qa\right)$$

The corrective term δq is described in Eq. (3), which shows that the effect of the charges of proximal neighbours is accounted as a sum of sums of partial charges multiplied by a bond reduction factor T that is exponentially decreased as the topological distance increases, i.e., the sum of the charges of the alpha neighbours is multiplied by T, the sum of the charges of the beta neighbours is multiplied by T^2^, and the sum of the charges of the gamma neighbours is multiplied by T^3^. The value of T is set to 0.274 and was taken from Gerber’s work. Note that alpha, beta, and gamma, represent the number of covalent bonds present between the atom of which the strength is calculated and a neighbouring atom (e.g., alpha identifies all atoms covalently linked to the atom in question; beta identifies all atoms that are two covalent bonds away from the atom in question).

3 $$\mathrm{\delta q}=\mathrm{T }\sum_{k}^{\alpha nbr}qk+ {\mathrm{T}}^{2} \sum_{k}^{\beta nbr}qk+ {\mathrm{T}}^{3} \sum_{k}^{\gamma nbr}qk$$

The polar contribution (ΔG^p^_hydr_) to the free energy of hydration is then calculated as described in Eq. (4), which consists of producing sums of atomic donor (sd_i_) and acceptor strengths (sa_i_) adjusted by their corresponding number of hydrogens (n_H_) and lone pairs (n_LP_) elevated by the exponential parameters exp_d_ and exp_a_. The sums of donor and acceptor strengths are then further corrected by the free parameters g_d_ and g_a_ and finally summed up to yield ΔG^p^_hydr_. The parameters exp_d_, exp_a_, g_d_ and g_a_, were set to 0.50, 0.34, 0.908, and −16.131, respectively. These parameters were determined by fitting against the data from Gerber’s work (See Model fitting and validation).

4 $${\Delta G}_{hydr}^{p}={g}_{d}\sum_{i}^{donors}{sd}_{i} {({n}_{H})}^{exp_\text d}+{g}_{a}\sum_{i}^{acceptors}{sa}_{i} {({n}_{LP})}^{exp_\text a}$$

### Apolar term

Our method calculates the apolar contribution (ΔG^a^_hydr_) to the free energy of hydration using the linear equation proposed by Gerber, as described in Eq. (5), using kallisto as an atomic featurizer. The apolar contribution consists of a constant term (g_0_), a surface term that incorporates a free parameter (g_s_) and the topological surface area (N_s_), a ring term that incorporates a free parameter (g_r_) and the ring count (N_r_), and two π-orbital dependent terms, each one incorporating a free parameter (g_π_^2^ and g_π_^1^), and the π-orbital count inside sp_k_-hybridized (k = 1, 2) atoms (N_π_^2^ and N_π_^1^).

5 $${\mathrm{\Delta G}}_{\mathrm{hydr}}^{\mathrm{a}}= {\mathrm{g}}_{0} + {\mathrm{g}}_{\mathrm{s}}{\mathrm{N}}_{\mathrm{s}} + {\mathrm{g}}_{\mathrm{r}}{\mathrm{N}}_{\mathrm{r}} + {\mathrm{g}}_\pi ^2{\mathrm{N}}_\pi ^2 + {\mathrm{g}}_\pi^1{\mathrm{N}}_\pi ^1$$

The topological surface area (N_s_) is calculated as a sum of atomic contributions as described in Eq. (6). Each contribution is calculated by incorporating the atomic van der Waals radius (r_i_^vdW^) as obtained by kallisto, the number of non-hydrogen ligands connected to each non-hydrogen atom (n^i^_l_), and a hybridization number (h^i^_sp1_ = 1, h^i^_sp2_ = 2, and h^i^_sp3_ = 3) as defined in Eq. (7).

6 $${\mathrm{N}}_{\mathrm{s}} = {\sum }_{\mathrm{i}}{N}_{s}^{i}$$

7 $${N}_{s}^{i}=4\uppi {\left({r}_{i}^{vdW}\right)}^{2}\left(1-\frac{{{n}^{i}}_{l}}{{h}_{spk}^{i}+1}\right)$$

The ring (N_r_) and both π-orbital counts (N_π_^2^ and N_π_^1^) are calculated using RDKit, where the π-orbital count is increased by two for sp_1_-hybridized atoms and by one for sp_2_-hybridized atoms. The parameters g_0_, g_s_, g_r_, g_π_^2^, and g_π_^1^, were set to 1.884, 0.0467, − 3.643, − 1.174, and − 1.602, respectively. These parameters were determined by fitting against the data from Gerber’s work (See Model fitting and validation).

### Interaction term

Our method reimplements the interaction contribution term (ΔG^i^_hydr_) originally described by Gerber. This empirical correction accounts for interactions between proximal hydrogen-bond acceptors (origin atoms) which may influence the free hydration energy of the molecule and is evaluated over their neighbours (n), their nearest-neighbours (nn), and their nearest-nearest neighbours (nnn) as described in Eq. (8), which includes atomic contributions and two free parameters (g_i_ and F). The atomic contributions are calculated as shown in Eq. (9) by multiplying the acceptor strength (sa) of a given atom by its number of lone pairs (n_LP_) elevated to the exponential parameter for hydrogen-bond acceptors (exp_a_). The parameters g_i_ and F were set to 4.9996 and 0.514, respectively. These parameters were determined by fitting against the data from Gerber’s work (See Model fitting and validation).

8 $${\mathrm{\Delta G}}_{\mathrm{hydr}}^{i} ={g}_{i}{\sum }_{j}{a}^{j}\left({\sum }_{k}^{n}{a}^{k}+{\sum }_{l}^{nn}{a}^{l} +F {\sum }_{m}^{nnn}{a}^{m}\right)$$

9 $${a}^{p}={sa}_{p}{\left({n}_{LP}^{p}\right)}^{exp_{a}}$$

### Advantages and limitations of the method

Our model, as for that of Gerber, describes the polar term of the hydration free energy as simply coming from the partial charges of atoms summed and adjusted by corrective factors, and the apolar term as a five-parameter equation derived from a small set of hydrocarbons. The solvent is not modelled; the conformational, steric, and intramolecular interaction effects are not accounted for; the interaction between proximal functional groups is only estimated empirically within the interaction term. In addition, donors and acceptors of hydrogen bonding are simply considered as atoms bonded to hydrogens or with one or more lone pairs, respectively, and the bond directionality is not modelled. These generalisations, however, come with some advantages: First, this logic allows the calculation of the free energy of hydration in centiseconds, enabling interactive design, analysis, or featurisation for more complex modelling techniques (e.g., machine learning); and second, the model includes the contributions of halogens as acceptors of hydrogen bonds, which can be used to understand further the relationship between compound structures and their activities/properties.

## Results and discussion

### Model fitting and validation

**Figure 1:**
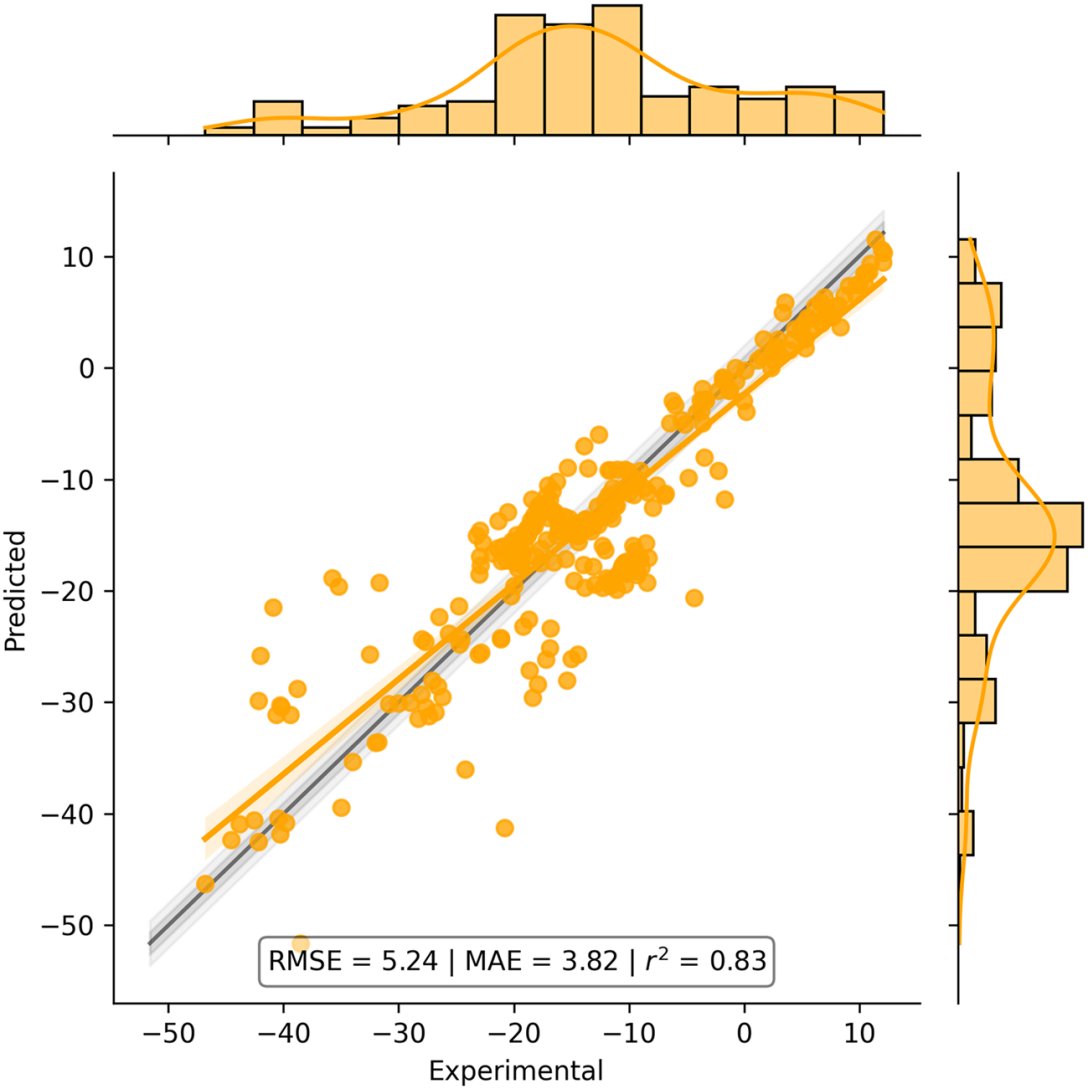
Correlation plot between predicted and experimental Hydration Free Energies from the data set described by Gerber. MAE and RMSE values are given in kJ mol^−1^.

The parameters used by Jazzy to calculate the Free Energy of Hydration were fitted using the experimental data from Gerber’s work. The correlation plot between predicted and experimental values is reported in Fig. 1 along with the mean absolute error (MAE), root mean squared error (RMSE), and the coefficient of determination (r^2^). Figure 1 shows that the model predicted very accurately compounds with free energies between − 10 and + 10 kJ mol^−1^, then the accuracy of the predictions becomes poorer as energies become more negative. The inspection of the results revealed that the model could predict with high accuracy molecules prevalently apolar or with only one or two polar groups. Compounds with higher flexibility, particular mesomeric systems, or groups that could interact with each other produced the lowest accuracies. An interesting example is that of the compounds 2-, 3-, and 4-nitro phenols which produced absolute errors of ~ 20, ~ 1.5, and ~ 2 kJ mol^−1^, respectively. Similar errors are also reported for Gerber’s method (~ 20, ~ 1, ~ 3.5 kJ mol^−1^). These results suggest that the implementation does not take into account the intramolecular interaction between the hydroxyl and nitro group in the 2-nitrophenol, for which both methods produced an error ten times higher compared to those of the 3- and 4- nitrophenols.

The metrics obtained for Jazzy (RMSE = 5.24 kJ mol^−1^, MAE = 3.82 kJ mol^−1^, r^2^ = 0.83) were compared to those from Gerber (RMSE = 4.07 kJ mol^−1^, MAE = 2.47 kJ mol^−1^, r^2^ = 0.90) to differentiate the two implementations: A potential explanation for the slightly worse metrics obtained for Jazzy could rely on the lack of the equation terms accounting for higher-order charge effects (dipole and quadrupole) which were not implemented in our method to increase its computational efficiency and decrease the model complexity (See “Implementation”). The errors produced by Jazzy and Gerber could not be further investigated due to the lack of error measures on the experimental data, which did not allow us to assess whether or not predictions produced reasonable deviations from their corresponding measured values. The prediction of the free energy of hydration of 292 compounds from Gerber’s data set took 20.4 s on a laptop (0.06 s per compound).

**Figure 2:**
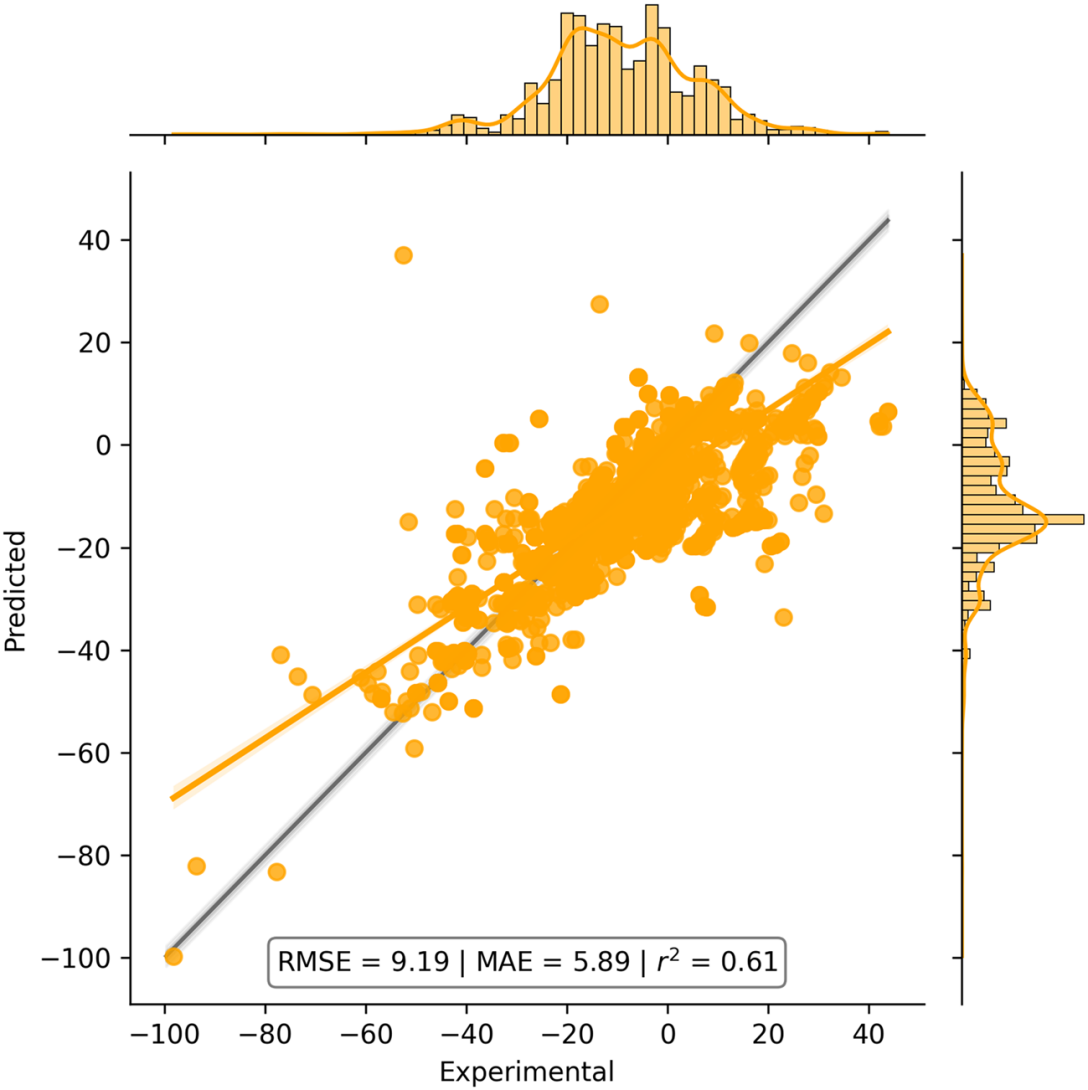
Correlation plot between predicted and experimental Hydration Free Energies from a subset of the Guthrie database. MAE and RMSE values are given in kJ mol^−1^.

The fitted model was validated externally against a subset of the Guthrie database of Free Energies of Hydration (GuthrieSolv). This validation was motivated by the need to determine whether the fitting of Jazzy produced overfit to the training data and to compare its errors to those from the experiments. Note that, the data set used in the external validation contains ten times (~ 3000) the number of data points used to fit Jazzy—with an average experimental error of 2.6 kJ mol^−1^ and a maximum of 10 kJ mol^−1^—and with a wider range of free energies from − 100 to + 40 kJ mol^−1^. The correlation plot between predicted and experimental values is reported in Fig. 2 with the same average metrics used in the fitting.

Figure 2 describes trends that are less clear compared to those reported for the validation against Gerber’s data as the accuracy of predictions remains similar across the entire interval of free energies. The inspection of the results revealed that Jazzy maintained high accuracy for compounds with groups not interacting with each other and rigid structures. A wider range in discrepancy between experimental and predicted values in this experiment is likely to be a consequence of the heterogenicity of the sources of data in the Guthrie set compared to that of Gerber which was created from the same data source. The compound that produced the highest errors were those containing phosphonate groups or long aliphatic chains and polar groups, i.e., compounds that are likely to form supramolecular aggregates in solution. The metrics from this validation (RMSE = 9.19 kJ mol^−1^, MAE = 5.89 kJ mol^−1^, r^2^ = 0.61) show that Jazzy still produced an average error in the range of one kcal mol^−1^ (4.2 kJ mol^−1^) although double than the average error from the experiments. The lack of public accessibility to Gerber’s method did not allow us to benchmark Jazzy against it for this data set. The prediction of the free energy of hydration of 3,313 compounds from Guthrie’s data set took 175.6 s on a laptop (0.05 s per compound).

### Hydrogen-bond donor strengths

Six cyclin-dependent kinase 2 (CDK2) inhibitors from Chen et al. which share the same aminothiazole scaffold were inspected using our method to rationalise the effect of their substituents on their binding affinity. The selection of these compounds was motivated by the presence of two hydrogen-bond donors interacting consistently with two key residues within the receptor which could be analysed on their strengths using Jazzy. The selection was also motivated by the availability of their protein–ligand complexes (PDB ligand IDs: X02, X35, X36, X44, 20Z, 26Z) in the Protein Data Bank (https://www.rcsb.org/), and their activities in ChEMBL (https://www.ebi.ac.uk/chembl/), which range from 15 to 0.07 nM. The compound structures, their identifiers, and activities are summarised in Table 1.

**Table 1:** The aminothiazole scaffold shared across the selected ligands, their R1 and R2 substituents, PDB ligand identifiers, and CDK2 pIC50s from ChEMBL.


PDB Ligand ID	R1	R2	pIC50 (M)
X02	Vinyl	Phenyl	4.82
X35	Vinyl	3-pyridine	5.51
X36	Phenyl	Phenyl	6.03
X44	4-Phenyl sulfonamide	4-Phenyl sulfonamide	7.15
20Z	4-Phenyl sulfonamide	2-Naphthalene	4.07
26Z	4-Phenyl sulfonamide	3-Aniline	7.15

The aminothiazole scaffold also describes the primary and secondary amine hydrogens responsible for the interaction with the residues E81 and L83, respectively. The interaction between these hydrogens and the CDK2 receptor is suggested to be shared across all the selected ligands^22^.

**Figure 3:**
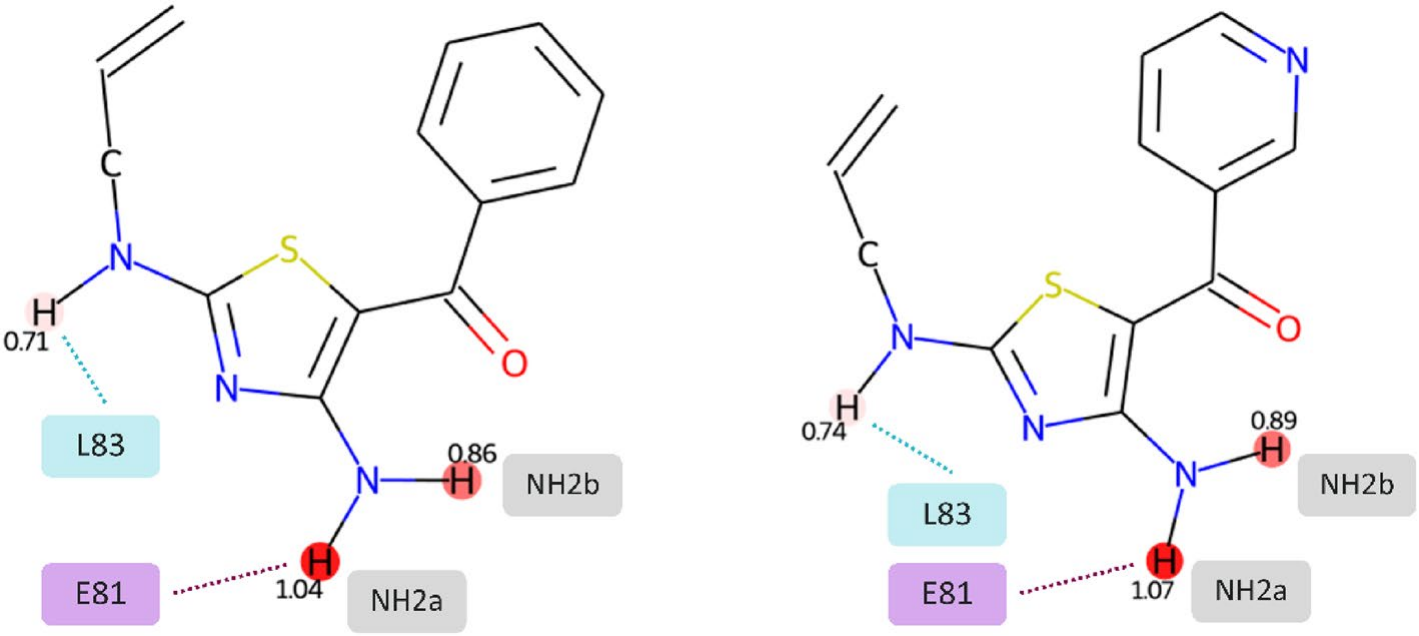
Atomic donor strength depictions for the compounds X02 (left) and X35 (right) produced by Jazzy. Donor strengths are annotated with their corresponding values and highlighted using a red gradient, where more intense colours indicate greater strengths. The difference in strength between the two symmetrical hydrogens (NH2a and NH2b) of each amine group denotes the ability of Jazzy of capturing the effect of conformations on the hydrogen-bond donor/acceptor strengths.

Jazzy was applied against the active conformations of the selected inhibitors to produce atomic and molecular hydrogen-bond strengths which are reported in Table 2. An example of atomic donor strength depiction produced by Jazzy is reported in Fig. 3 for the compounds X02 and X35. The atomic strengths are also reported as image renderings for each individual molecule in the Supplementary Information.

**Table 2:** Atomic and molecular strengths calculated using Jazzy against the active conformations of the selected inhibitors: sd_NH2a-E81_ and sd_NH2b_ refer to the hydrogens of the primary amine, where NH2a-E81 interacts with the residue E81 and NH2b does not; sd_NH-L83_ refers to the hydrogen of the secondary amine; sdx_mol_ is the sum of X–H donor strengths where X is any non-carbon atom; sdc_mol_ is the sum of C–H donor strengths; sd_mol_ is the sum of X–H donor and C–H donor strengths.

Ligand	sd_NH2a-E81_	sd_NH2b_	sd_NH-L83_	sdx_mol_	sdc_mol_	sd_mol_
X02	0.86	1.04	0.71	2.61	5.34	7.95
X35	0.89	1.07	0.74	2.70	5.49	8.18
X36	0.84	1.03	0.71	2.59	5.60	8.19
X44	0.98	1.14	0.82	6.96	6.75	13.71
20Z	0.92	1.09	0.76	4.67	7.31	11.98
26Z	0.96	1.12	0.88	6.28	5.59	11.86

The atomic strengths were specifically generated for the hydrogens involved in the interactions with the residues E81 (sd_NH2a-E81_) and L83 (sd_NH-L83_) which Chen et al. suggested to be preserved across the ligands using molecular dynamics. Table 2 also includes the strengths of the hydrogen on the primary amine that is not interacting with E81 (sd_NH2b_).

The differences in values for the two symmetric hydrogens (sd_NH2a-E81_ and sd_NH2b_) on the primary amine are due to the implementation of Jazzy: Strengths are calculated from partial charges that depend on atomic electronegativities scaled by the coordination numbers of donors/acceptors and their neighbours, which in turn are impacted by the proximity of other atoms in the molecule as described by Caldeweyher and colleagues^20^. This feature allows Jazzy to capture the effect of conformations on the individual hydrogen-bond donors/acceptors in a molecule, which can potentially be used in combination with other scoring methods to discriminate between active from inactive conformers.

The pIC50s and strengths from Tables 1 and 2 were correlated to yield the correlation coefficients ‘r’ in Table 3, which show moderate and strong positive correlations between the inhibition of CDK2, the strengths of the hydrogens NH2a-E81, NH-L83, and the molecular X–H donor strength sdx_mol_, respectively. Slightly less positive correlations are found for NH2b and the total molecular donor strength sd_mol_, and finally, a negative correlation is reported for the molecular C–H donor strength sdc_mol_. Given these results, a qualitative inspection was carried out to get more insights into the effect of the substituents structures: For example, the increase in activity between X02 and X35 can depend on the replacement of the phenyl substituent with a pyridine, which due to its electron-withdrawing effect, may increase sd_NH2a-E81_, sd_NH2b_, and sd_NH-L83_. The increase in activity between X02 and X36 can be related to an increase in the hydrophobic surface from the replacement of the vinyl group with a phenyl since sd_NH-L83_ and sdx_mol_ are almost the same for those compounds.

**Table 3:** Correlation coefficients ‘r’ calculated between pIC50s and hydrogen-bond donor strengths for the individual hydrogens (NH2a-E81, NH2b, NH-L83) and the molecule by accounting for only X–H (sdx_mol_), only C–H (sdc_mol_), or both (sd_mol_).

Correlation coefficients ‘r’					
r_pIC50/sdNH2a-E81_	r_pIC50/sdNH2b_	r_pIC50/sdNH-L83_	r_pIC50/_sdx_mol_	r_pIC50/_sdc_mol_	r_pIC50/_sd_mol_
0.59	0.53	0.69	0.60	− 0.21	0.39

The formula used for the calculation of ‘r’ is reported in the Supplementary Information.

The decrease in activity between 26Z and 20Z could be explained by the greater steric hindrance of the naphthalene substituent over the sulfonamide—although the decrease of sd_NH-L83_ could also be an indicator of lower affinity with the target. These results are in agreement with those of Chen et al., and suggest that X–H donor strengths, in particular those of the hydrogens interacting with E81 and L83, can potentially be used to rationalise existing data and identify more active compounds against CDK2. However, the number of data points (i.e., sample size) on this molecular series may not be sufficient to underpin the dominance of hydrogen bonding over other types of interactions as well as to provide prospective information on the interaction between other chemical series and the CDK2 receptor.

### Hydrogen-bond acceptor strengths

A similar experiment to that described in the previous section was applied to the 11β-hydroxysteroid dehydrogenase type 1 (11β-HSD1) inhibitors from Robb et al. These inhibitors share the same pyrazolo[1,5-a]pyrimidine scaffold and were designed by modulating the strengths of two hydrogen-bond acceptors to optimise their potency and other properties of pharmaceutical interest. In particular, Robb and colleagues designed these compounds using a quantum mechanics method based on the calculated molecular electrostatic potential, given the knowledge a priori of the compounds’ binding mode and key hydrogen-bonding interactions. The selection of these compounds for our experiment was motivated by the availability of a greater number of data points compared to those from Chen et al. and the presence of hydrogen-bond acceptors responsible for shifts in bioactivity that could be analysed using Jazzy. Specifically, the acceptors are the nitrogen in position 1 of the pyrazole ring, which produces an intermolecular interaction with the residue G216, and its γ-neighbour, the nitrogen on the pyrimidine ring, which is involved in an intramolecular hydrogen bonding that can stabilise the compounds into their bioactive conformations, hence affecting their potency. The compound structures, identifiers, and activities are summarised in Table 4.

**Table 4:** The pyrazolopyrimidine scaffold shared across the selected ligands, their R1, R2, and R3 substituents, ligand identifiers and 11β-HSD1 pIC50s from Robb et al.


Ligand ID	R5	R6	R7	pIC50 (M)
2	H	H	H	7.0
3^a^	H	H	H	6.0
4	CH3	H	H	7.7
5	H	CH3	H	7.2
6	H	H	CH3	7.5
7	CH3	H	CH3	8.2
8	CH3	H	CH2OCH3	7.6
9	CH3	H	CHF2	7.6
10	CH3	H	CF3	7.3
11	H	H	CF3	6.7
12	Cyclopropyl	H	CHF2	8.0
13	CH2OCH3	H	CH3	7.8
14	H	CH2CH3	H	7.5
15	H	Cl	H	7.3
16	H	Cyano	H	6.6
17	H	OCH3	H	7.2
18	H	CH2CH2OH	H	6.5

The pyrazolopyrimidine scaffold also describes the intramolecular (int) and intermolecular (ext) hydrogen-bond acceptors. ^a^The compound contains an imidazo-pyridazine scaffold, which is different to the others in the analysis but sufficiently similar to be included.

Jazzy was applied to the MMFF94 energy-minimised conformations of these compounds due to the lack of availability of their active conformations. Atomic strengths were generated for the nitrogen atoms involved with the intra- and intermolecular hydrogen bonds. The strengths produced by our method and those from Robb et al.^23^ are reported in Table 5.

**Table 5:** Atomic and molecular strengths calculated using Jazzy against energy-minimised conformations of the selected inhibitors (sa_int_, sa_ext_, sa_mol_) and the acceptor strengths calculated by Robb and colleagues (Log kβ_int_, Log kβ_ext_).

Ligand	Log kβ_int_	sa_int_	Log kβ_ext_	sa_ext_	sa_mol_
2	1.56	0.68	1.49	0.63	4.36
3	0.54	0.57	2.58	0.70	4.20
4	1.64	0.69	1.70	0.64	4.36
5	1.66	0.72	1.68	0.63	4.40
6	1.84	0.78	1.42	0.66	4.50
7	1.90	0.78	1.62	0.66	4.55
8	1.82	0.68	1.46	0.68	5.25
9	1.20	0.76	0.70	0.48	4.99
10	0.98	0.65	1.34	0.49	5.04
11	0.88	0.61	1.11	0.48	5.01
12	0.85	0.68	0.81	0.51	5.00
13	1.69	0.66	1.70	0.67	5.14
14	1.69	0.73	1.70	0.65	4.48
15	0.92	0.76	1.09	0.59	4.60
16	0.41	0.63	0.42	0.59	4.90
17	1.56	0.66	1.68	0.62	5.02
18	1.84	0.67	1.97	0.64	5.28

Internal (int) and external (ext) strengths represent those associated with the intra- and intermolecular hydrogen bonds, respectively. sa_mol_ represents the sum of all atomic acceptor strengths.

The pIC50s and strengths from Tables 4 and 5 were correlated to yield the coefficients in Table 6, which shows strong positive correlations between the inhibition of 11β-HSD1 and internal strengths; weaker negative correlations between inhibition and external strengths; and a slight positive correlation for the molecular acceptor strength. These results are in agreement with those from Robb and colleagues which suggest that the intramolecular hydrogen bond in this compound series plays the role of biasing the compounds towards assuming their active conformations over a potential inactive ensemble, hence reducing the enthalpic penalty that affects their bioactivity in solution. Our results also align in suggesting that increasing the strength of the intermolecular hydrogen bond produces a negative effect on the activity against 11β-HSD1 as the formation of such a hydrogen bond may result in a larger desolvation penalty. The agreement between our method and the calculations produced by Robb et al. and the consensus produced by the correlation of a higher number of data points suggest that Jazzy can produce meaningful estimations for both intra- and intermolecular hydrogen-bond modelling.

**Table 6:** Correlation coefficients ‘r’ calculated between pIC50s and hydrogen-bond acceptor strengths generated by Jazzy (sa_int_, sa_ext_, sa_mol_) and Robb and colleagues (Log kβ_int_, Log kβ_ext_) for the acceptor nitrogens of the pyrazolopyrimidine.

Correlation coefficients ‘r’				
rpIC50/Log kβ_int_	rpIC50/sa_int_	rpIC50/Log kβ_ext_	rpIC50/sa_ext_	rpIC50/sa_mol_
0.48	0.67	− 0.27	− 0.08	0.10

The formula used for the calculation of ‘r’ is reported in the Supplementary Information.

## Conclusions and future outlook

We have implemented an open-source tool referred to as Jazzy that allows the fast calculation of hydrogen-bond strengths, either at the atomic or molecular level, and hydration free energies. We have reported the implementation of our method, its parameter fitting, and validation against two data sets of experimentally measured free energies of hydration. We have also shown that our method compares to that of Gerber and pointed out its strengths and limitations. Jazzy can also produce depictions of compounds and their strengths in real time to enable the elucidation of compound SAR/SPR in contexts where hydrogen bonding is known to play a critical role. We have demonstrated such an application by running Jazzy against a chemical series of CDK2 inhibitors, and have shown that our method can be used to understand the relationship between experimental activities and hydrogen-bond donor strengths. We have also described a similar experiment on a series of 11β-HSD1 inhibitors, rationally designed by a team of chemists by modulating the strengths of two hydrogen-bond acceptors using quantum mechanics, and shown that Jazzy could produce correlated strengths for both intra- and intermolecular acceptors. While the simplistic approach implemented in Jazzy may not allow capturing solvent and intramolecular effects or the tendency to form supramolecular architectures, results suggest that it can be used as an alternative tool to computationally intensive methods for interactive design, screening, or machine-learning featurisation. With regard to these applications, we strongly believe the selection of an open-source license for Jazzy will promote its adoption, enable improvements of the method, and deliver value in the field of molecular modelling.

## Methods

### Model fitting and validation

The fitting and validation against Gerber’s data were carried out as follows: The IUPAC names and free energy measures were obtained from the original paper by Gerber. Names were converted into SMILES using the Chemical Identifier Resolver by CACTUS^26^. SMILES were read using RDKit, hydrogens were added, coordinates were initialised using a fixed seed, and conformations were minimised using the MMFF94 force-field method implemented in RDKit. Protonation states, tautomeric forms, and major species in water for the selected compounds were not evaluated in this experiment due to the lack of open-source tools for such a purpose, and to allow direct comparison with the results from Gerber’s validation. Jazzy was then applied to yield the predicted free energies. Experimental and predicted free energies were used to produce the mean absolute error as a loss measure for the model. The parameter fitting was operated by the Optuna framework^27^ implementing an early stop policy after 300 cycles of no improvement. The best model’s parameters are reported in the Implementation.

The validation against Guthrie’s data was performed as follows: The complete database was obtained from Guthrie et al. then a data set was created from it by selecting only compounds associated with measures in the units of kJ mol^−1^ or kcal mol^−1^. The excluded measures were expressed in heterogeneous units including M/atm and Pascal. In addition, all compounds described in Gerber’s data were removed from the set. A series of histograms reporting the distribution of a selection of molecular descriptors is included in the Supplementary Information. The SMILES in the resulting data set were then processed as described in the validation using Gerber’s data to produce the predicted free energies using the parameters obtained from the fitting.

### Hydrogen-bond donor strengths

Individual SD files were obtained from the Protein Data Bank for the ligands X02, X35, X36, X44, 20Z, and 26Z. The ligand files were read using RDKit, and hydrogens were added to them by preserving the ligand active conformations. Jazzy was then run against the ligands without minimising their conformational energies to produce molecular strengths and atomistic strength depictions. Atomic donor strengths were calculated as described in Eq. (1). Molecular strengths were calculated by summing up all the atomic donor strengths.

### Hydrogen-bond acceptor strengths

The IUPAC names of the compounds described by Robb et al. were obtained from the literature and converted into SMILES strings using the Chemical Identifier Resolver by CACTUS^28^. SMILES were read using RDKit, hydrogens were added, coordinates were initialised using a fixed seed, and conformations were minimised using the MMFF94 force-field method implemented in RDKit. Jazzy was then run against the minimised ligands to produce their atomic acceptor strengths as described in Eq. (2). Molecular strengths were calculated by summing up all the atomic acceptor strengths multiplied by their corresponding number of lone pairs.

## Supplementary Information

Supplementary Information.

## Data Availability

The data used to train and validate Jazzy, its source code, and Jupyter notebooks containing the experiments described in this manuscript, are freely available without any restriction on GitHub https://github.com/AstraZeneca/jazzy. Project name: Jazzy. Project home page: https://github.com/AstraZeneca/jazzy, https://pypi.org/project/jazzy/. Operating system(s): Linux, macOS, and Windows. Programming language: Python. License: Apache License 2.0.

## Abbreviations

11β-HSD1: 11β-Hydroxysteroid dehydrogenase type 1
CACTUS: CADD group chemoinformatics tools and user services
CDK2: Cyclin-dependent kinase 2
EMBL: European molecular biology laboratory
IUPAC: International union of pure and applied chemistry
MAE: Mean absolute error
MD: Molecular dynamics
MMFF94: Merck molecular force field 94
PDB: Protein data bank
RMSE: Root-mean-square error
SAR/SPR: Structure–activity/property-relationship
SMILES: Simplified molecular input line entry system
