# Supplementary material for: Fast calculation of hydrogen-bond strengths and free energy of hydration of small molecules
Source: Sci Rep. 2023 Mar 13;13:4143. doi: 10.1038/s41598-023-30089-x (PMC10011384; doi:10.1038/s41598-023-30089-x)

Supplementary Information

# Atomic hydrogen-bond donor strengths of the CDK2 inhibitors from Chen et al. (2018)

| 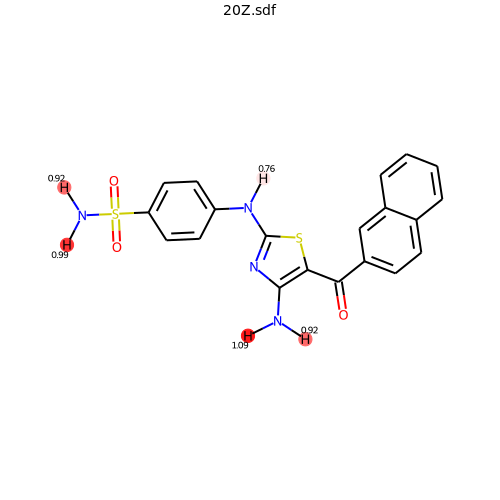 | 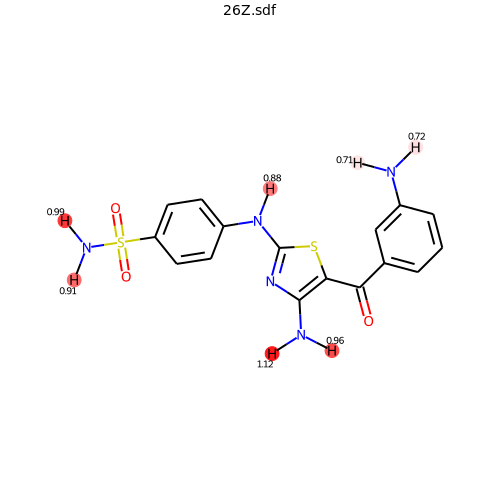 |
| --- | --- |
| 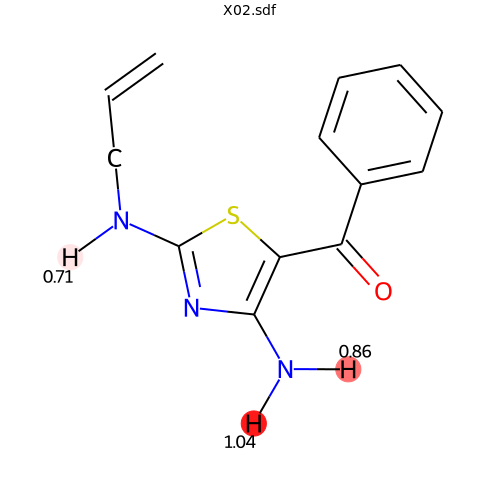 | 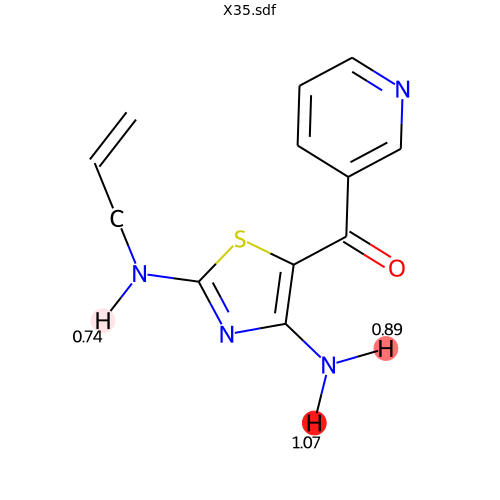 |
| 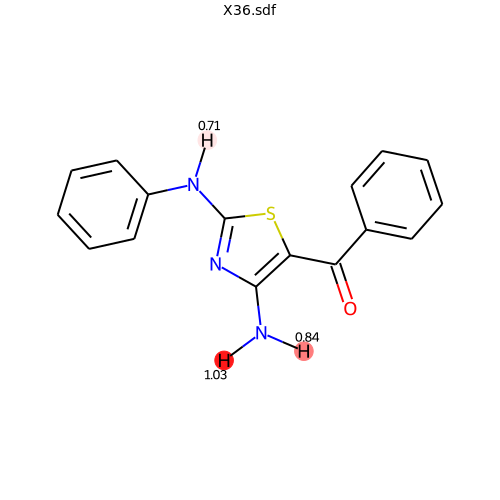 | 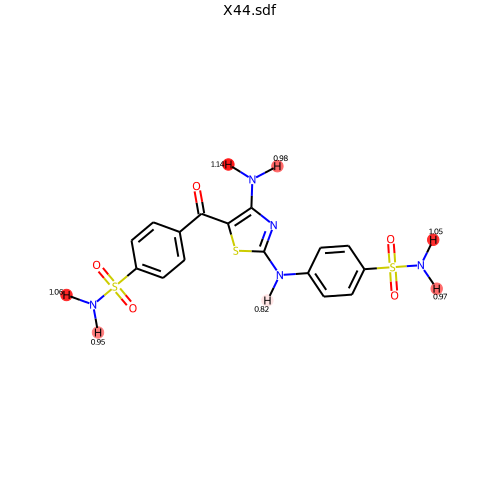 |

# Atomic hydrogen-bond acceptor strengths of the 11β-HSD1 inhibitors from Robb et al. (2015)

| 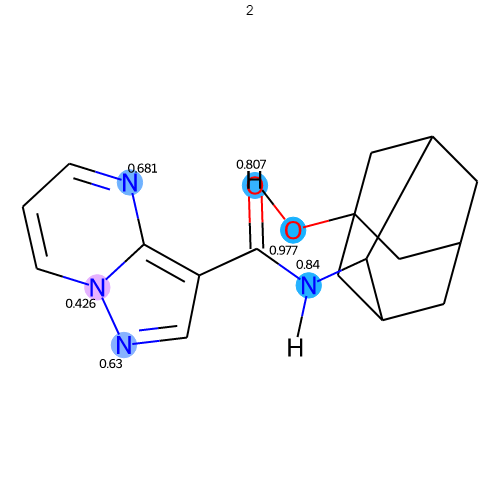 | 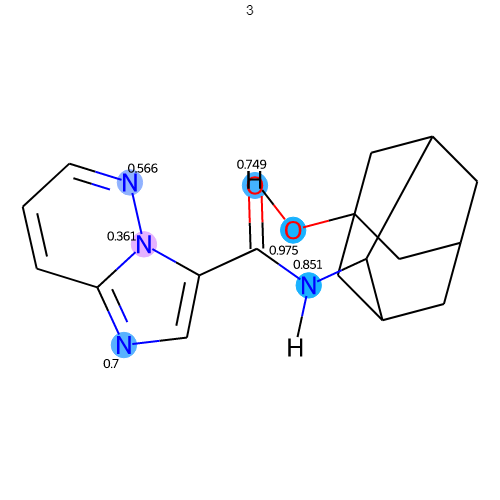 |
| --- | --- |
| 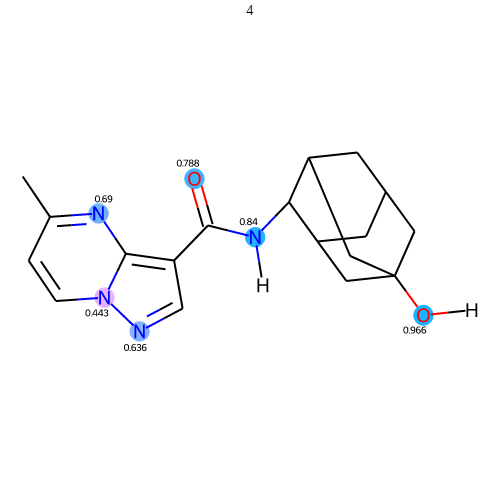 | 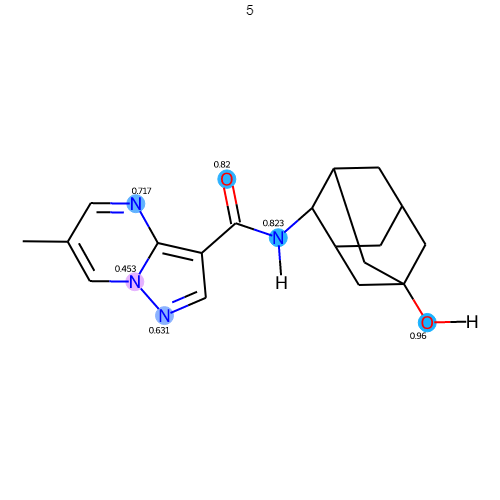 |
| 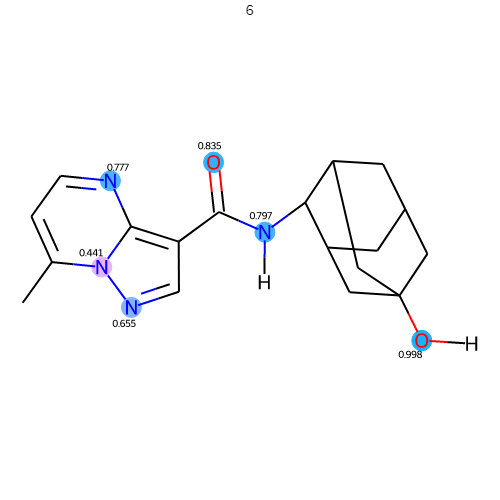 | 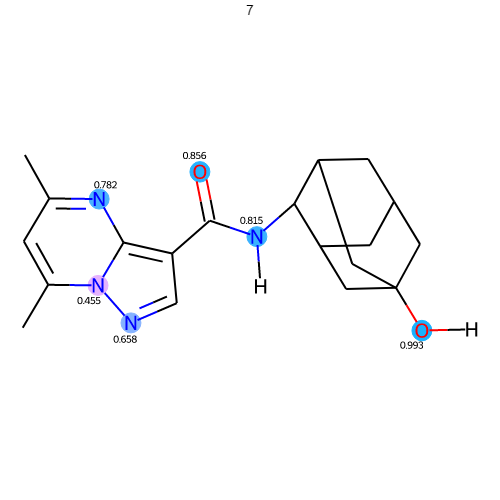 |
| 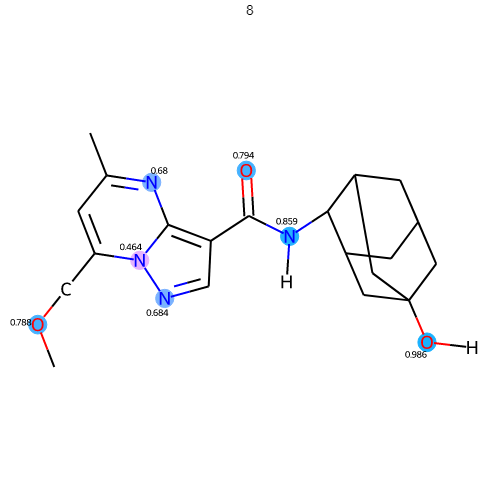 | 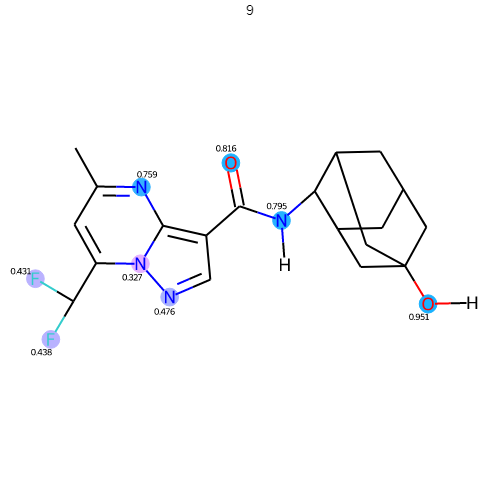 |
| 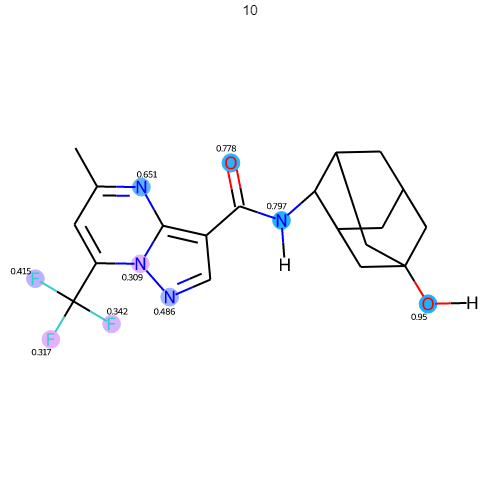 | 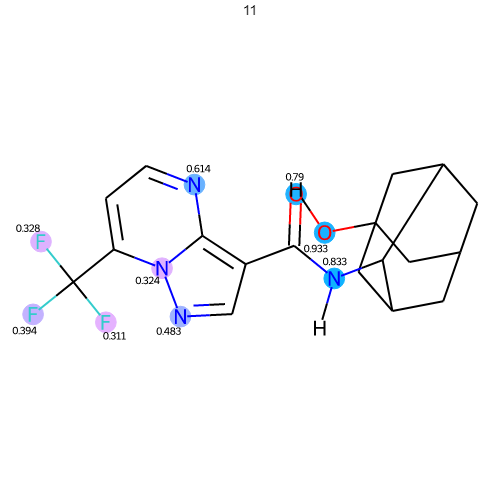 |
| 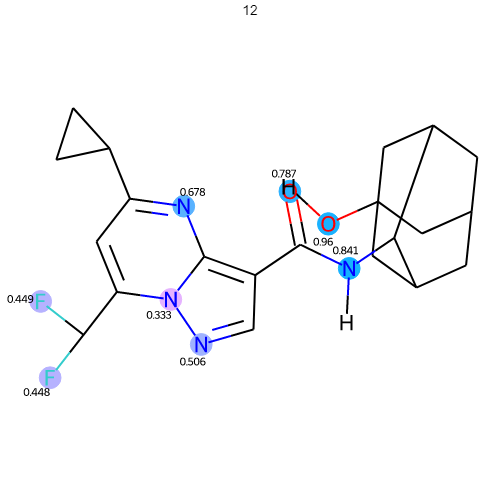 | 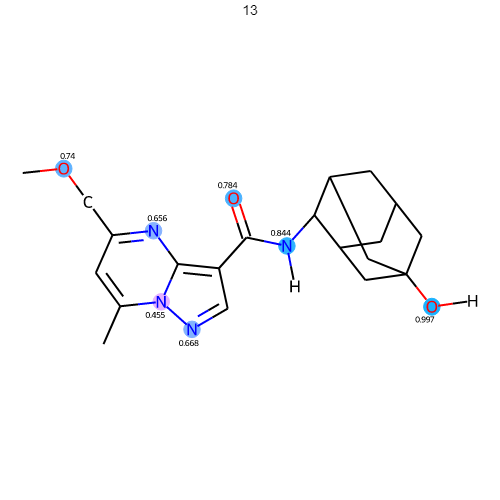 |
| 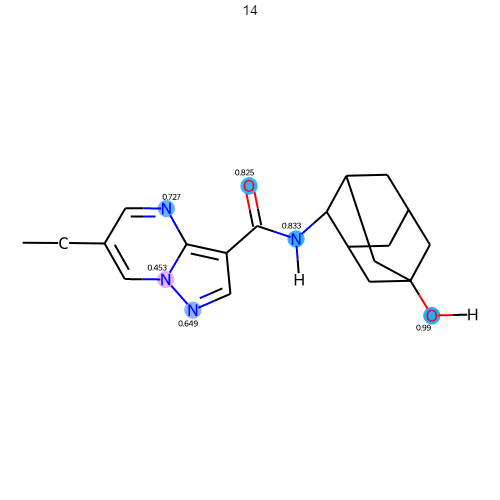 | 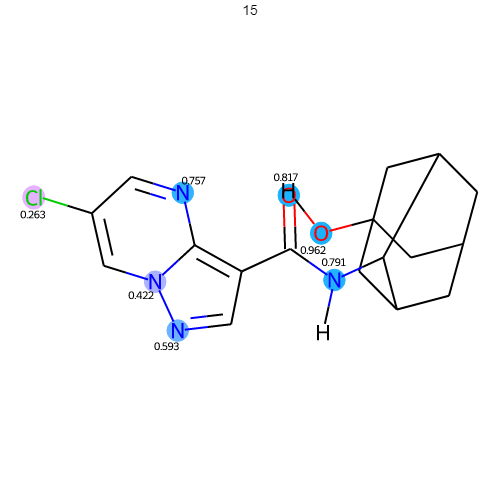 |
| 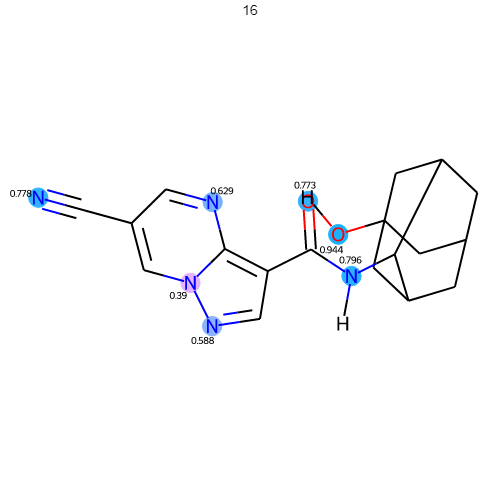 | 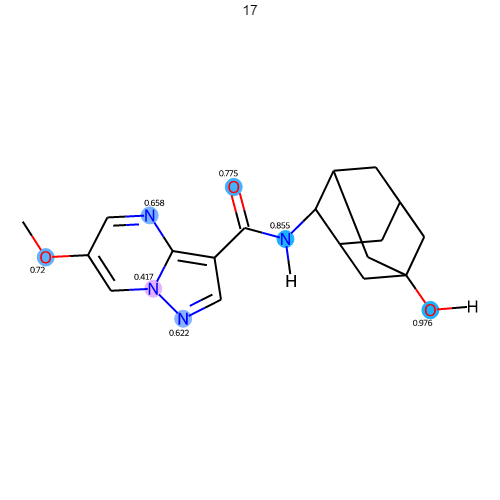 |
| 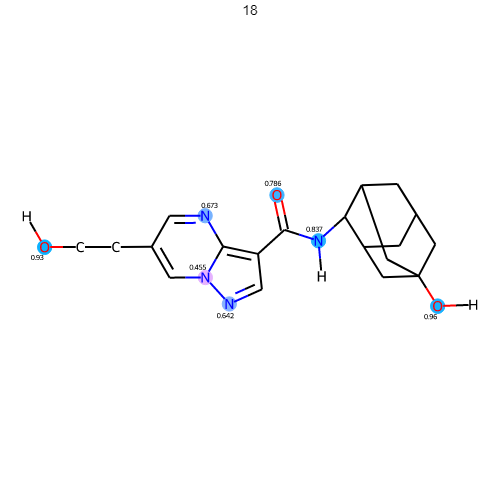 |  |

# Correlation coefficient ‘r’

$$r= \frac{\sum\left( x_{i}-\bar{x} \right)\left( y_{i}-\bar{ȳ} \right)}{\sqrt{\sum\left( x_{i}-\bar{x} \right)^{2}\sum\left( y_{i}-ȳ \right)^{2}}}$$

# Guthrie curated data set property distributions


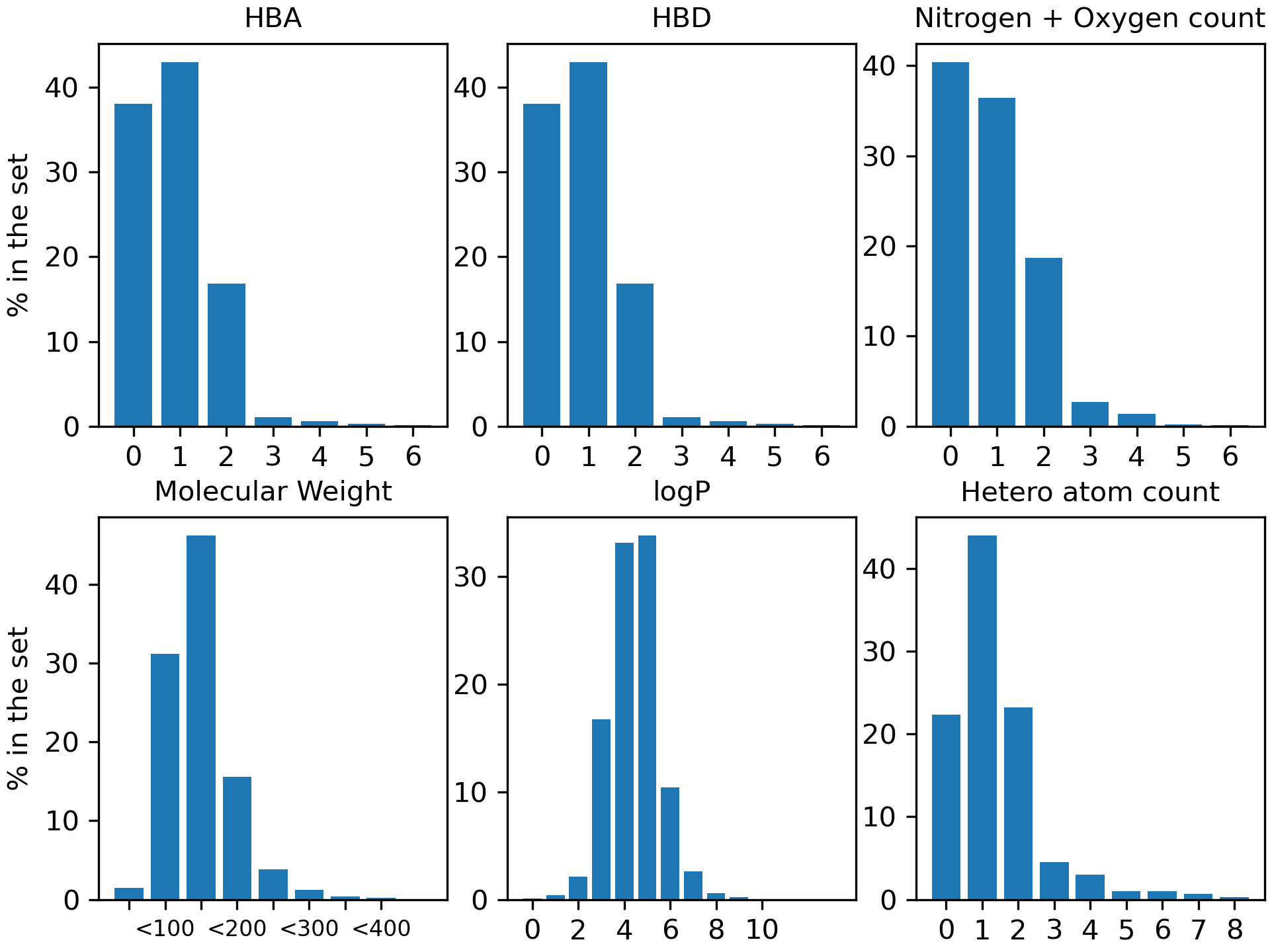

Supplement: Supplementary file 1 — Supplementary Information. [file 41598_2023_30089_MOESM1_ESM.docx]
